# *Abca7* deletion does not affect adult neurogenesis in the mouse

**DOI:** 10.1042/BSR20150308

**Published:** 2016-03-16

**Authors:** Hongyun Li, Tim Karl, Brett Garner

**Affiliations:** * Illawarra Health and Medical Research Institute, University of Wollongong, Wollongong, NSW 2522, Australia; † School of Biological Sciences, University of Wollongong, Wollongong, NSW 2522, Australia; ‡ Neuroscience Research Australia, Randwick, NSW 2031, Australia; § School of Medical Sciences, University of New South Wales, Sydney, NSW 2052, Australia; ║ Schizophrenia Research Institute, Randwick, NSW 2031, Australia

**Keywords:** ABCA7, Alzheimer's disease, bromodeoxyuridine, dentate gyrus, doublecortin, neurogenesis, subventricular zone

## Abstract

ATP-binding cassette transporter A7 (ABCA7) is highly expressed in the brain. Recent genome-wide association studies (GWAS) have identified *ABCA7* single nucleotide polymorphisms (SNPs) that increase Alzheimer's disease (AD) risk, however, the mechanisms by which ABCA7 may control AD risk remain to be fully elucidated. Based on previous research suggesting that certain ABC transporters may play a role in the regulation of neurogenesis, we conducted a study of cell proliferation and neurogenic potential using cellular bromodeoxyuridine (BrdU) incorporation and doublecortin (DCX) immunostaining in adult *Abca7* deficient mice and wild-type-like (WT) littermates. In the present study counting of BrdU-positive and DCX-positive cells in an established adult neurogenesis site in the dentate gyrus (DG) indicated there were no significant differences when WT and *Abca7* deficient mice were compared. We also measured the area occupied by immunohistochemical staining for BrdU and DCX in the DG and the subventricular zone (SVZ) of the same mice and this confirmed that ABCA7 does not play a significant role in the regulation of cell proliferation or neurogenesis in the adult mouse.

## Abstract (precis)

ATP-binding cassette transporter A7 (ABCA7) is expressed in the brain and linked with Alzheimer's disease. Since other ABC transporters regulate adult neurogenesis, we assessed neurogenesis in wild-type (WT) and *Abca7* deficient mice. *Abca7* deletion did not affect adult neurogenesis in the mouse.

## INTRODUCTION

ATP-binding cassette transporter A7 (ABCA7) is a member of the “A” subfamily of ATP-binding cassette transporters that were initially characterized by their capacity to transport lipids and other lipophilic molecules across membranes [1,2]. ABCA7 is expressed in the brain where it appears to be predominantly localized in microglia and, to a lesser degree, in neurons and oligodendrocytes [3]. Several lines of evidence implicate a role for ABCA7 in the regulation of Alzheimer's disease (AD) risk and amyloid pathology. Data from human genome-wide association studies (GWAS) indicate *ABCA7* is a risk factor for late-onset AD [4–9]. Additional genetic studies confirm the important association of *ABCA7* single nucleotide polymorphisms (SNPs) and methylation changes with AD [7–9], and recent work highlights that loss-of-function *ABCA7* variants confer increased AD risk [10]. This is in agreement with data showing that the AD-associated *ABCA7* SNP (rs3764650 that does not result in an amino acid change) is associated with reduced *ABCA7* expression levels in AD cases [11].

*In vitro* studies revealed that ABCA7 transfection into cell lines constitutively expressing human amyloid-β precursor protein (APP) results in a significant reduction in production of amyloid-β peptide (Aβ) peptides [12], whereas siRNA-mediated knockdown of ABCA7 increased Aβ production under similar experimental conditions [13]. In addition, phagocytic clearance of oligomeric Aβ was found to be impaired in ABCA7-deficient macrophages compared with wild-type (WT) macrophages [14]. In general agreement with these cell culture studies, work in AD mouse models has also provided evidence supporting a role for ABCA7 in amyloid homoeostasis. In the J20 AD transgenic mouse model, deletion of ABCA7 led to a doubling of insoluble Aβ levels and amyloid plaques in mice assessed at 17 months of age [14]. An independent study using the same *Abca7* null (*Abca7*^−/−^) mouse line but crossed with the TgCRND8 AD mouse model revealed an increase in the area of dense plaques at 18 weeks of age that the authors concluded was likely to be a reflection of the increased production of Aβ in the TgCRND8-*Abca7*^−/−^ mice [13]. It should be noted that even in the absence of the AD transgene expression, *Abca7*^−/−^ null mice were found to exhibit an age-dependent increase in the levels of the pathogenic (endogenous) Aβ42 peptide in the brain; assessed up to 6 months of age [13].

Although it is clear that ABCA7 has the capacity to regulate amyloidogenesis in mice, an entirely different question that has not been addressed centres on the potential impact that ABCA7 loss of function may have on adult neurogenesis. Our rationale for asking this question is based on previous studies showing that other ABC transporters including two that, like ABCA7, are also from the “Class-A” subfamily (i.e. ABCA2, ABCA3) appear to play a role in neurogenesis [15,16]. These studies along with the extensive literature highlighting the importance of adult neurogenesis in AD (see [17–20], for recent comprehensive reviews of this area) prompted our present study in which we have assessed cellular proliferation and neurogenesis in the dentate gyrus (DG) and subventricular zone (SVZ) of both WT and *Abca7*^−/−^ adult male mice.

## MATERIALS AND METHODS

### Animals

*Abca7*-deficient mice were generated and genotyped as described previously [21]. All mice were on a C57BL/6j background and backcrossed >15 times. Test mice were males at ∼8.5 months of age (WT, *n*=7, 251±7 days old; *Abca7*^−/−^, *n*=7, 256±9 days old). The animals we refer to as “WT” control mice were littermates derived from the same breeders that were used to generate the *Abca7*^−/−^ animals (i.e. both test cohorts were from *Abca7*^+/−^ breeders). Animal ethics approval was from the University of Wollongong Animal Ethics Committee.

### 5-Bromo-2-deoxyuridine injection, tissue preparation and immunohistochemistry

5-Bromo-2-deoxyuridine (BrdU, Sigma, B5002) was administered via intraperitoneal injection (i.p.) at a dose of 50 mg/kg twice a day (8 a.m. and 8 p.m.) for three consecutive days prior to euthanasia of animals. The animals were killed by methoxyflurane inhalation 4 h after the final BrdU injection and perfused with 0.9% NaCl followed by 4% paraformaldehyde (PFA). Tissues were preparation as described previously [22]. In brief, brains were removed and post-fixed in phosphate-buffered 4% PFA at 4°C for 16 h then cryoprotected in 30% (w/v) sucrose in 0.1 M phosphate buffer for 48 h. One hemibrain was coronally sectioned at 40 μm with a cryostat (CM1950, Leica Microsystems), the sections were collected in six series and stored at −20°C in cryoprotectant (3:3:4, glycerol:ethylene glycol:0.1 M phosphate buffer, v/v/v) for histological or immunohistochemical analysis. Every seventh hemibrain section was used for neurogenesis detection as described previously [23,24]. Briefly, after cryoprotectant removal, the sections were post-fixed with Bouin-4 [4% (w/v) PFA, 1% (w/v) picric acid and 5% (v/v) acetic acid, to improve nuclear antigen unmasking] for 15 min, then processed in sodium borohydride (0.1 mg/ml in 0.01 M PBS, to remove excess reactive aldehyde groups), denaturation (2 M HCl, 22°C, 1 h, then neutralization with 0.1 M borate buffer, pH 8.5) and H_2_O_2_ quenching [1% (v/v) H_2_O_2_ in PBS, 22°C, 30 min to quench endogenous peroxidase activity]. After blocking in Vector MOM reagent (Vector), the sections were stained with rat monoclonal anti-BrdU (0.5 μg/ml; Abcam, AB6326, from Sapphire Bioscience Pty Ltd) and biotinylated with goat-anti-rat IgG (1:200, Vector Laboratories), followed by Vectastain Elite ABC reagent (Vector Laboratories) or Streptavidin-HRP (Sigma, S2438 1:2000) and staining was visualized with ImmPact diaminobenzidine (DAB) peroxidase substrate (Vector Laboratories). To confirm the specificity of primary antibodies, control experiments were performed where sections were incubated for 16 h in the absence of primary antibody. Doublecortin (DCX), a commonly used marker for neuroblast cells [25,26] in the adult brain, was detected using rabbit anti-DCX (1:2000, Abcam, Ab18723, from Sapphire Bioscience Pty Ltd) as described as above for the BrdU protocol except the denaturation and MOM blocking steps were omitted and the secondary antibody used was biotinylated goat-anti-rabbit IgG (Sigma, B7389, 1:2000). All the staining procedures were carried out within a single immunostaining session to minimize variability.

### Image acquisition and quantification

Immunohistochemically stained sections were captured using a Scanscope XT Image scanner at 20× (Scanscope Console V10.2.0352, Aperio Digital Pathology System, Aperio Technologies). The images were exported in tiff format from Imagescope software (Aperio Technologies), and analysed using ImageJ software (http://rsbweb.nih.gov/ij/) using an established quantitative method [27]. Representative images were assembled with Photoshop CS2 for presentation in the figures. The numbers of BrdU +ve and DCX +ve cells in the DG were directly counted in the immunostained sections (every seventh coronal section, five sections per mouse) in a blinded manner under 20× magnification. This was achieved using tiff files generated from the Scanscope XT Image scanned images and the data were expressed as positive cell number per mm^2^. Due to the clustering of BrdU +ve cells in the SVZ and the overlap of neuronal structures in SVZ DCX staining, it was impractical to count absolute cell numbers and therefore the data were only acquired as positive stained occupied area (μm^2^) for this region of interest in each section (as described below).

To quantify BrdU +ve and DCX +ve staining, five coronal sections per brain were collected, 240 μm apart, grossly corresponding to sections 1.2–0.0 mm anterior to the Bregma (+1.2–0.0) according to the mouse brain atlas [28], were selected to measure the area occupied by positive staining in a defined region of interest area of the SVZ, i.e., the entire lateral wall of SVZ from corpus callosum to the anterior commissure (selected area: approximately 0.8–1.2 mm^2^, mean ± S.E.M., 0.94 ±0.07 mm^2^); and for measurement of hippocampal neurogenesis, five coronal sections spanning from 1.7 to 2.7 mm posterior to the Bregma (−1.7 to −2.7) were chosen to analyse the area occupied by positive staining in the known neurogenic region, i.e. the DG. This region includes the molecular layer, outer granule cell layer, subgranular zone (the most neurogenic region of the hippocampus) and hilus in the hippocampus formation (selected area approximately 0.8–1.2 mm^2^, mean ± S.E.M.: 0.93±0.11 mm^2^).

### Statistical analysis

Quantitative data are presented as mean ± S.E.M. (represented by the error bars). Statistical differences were assessed using the Student's *t* test where *P*<0.05 was considered significant.

## RESULTS AND DISCUSSION

**Figure 1:**
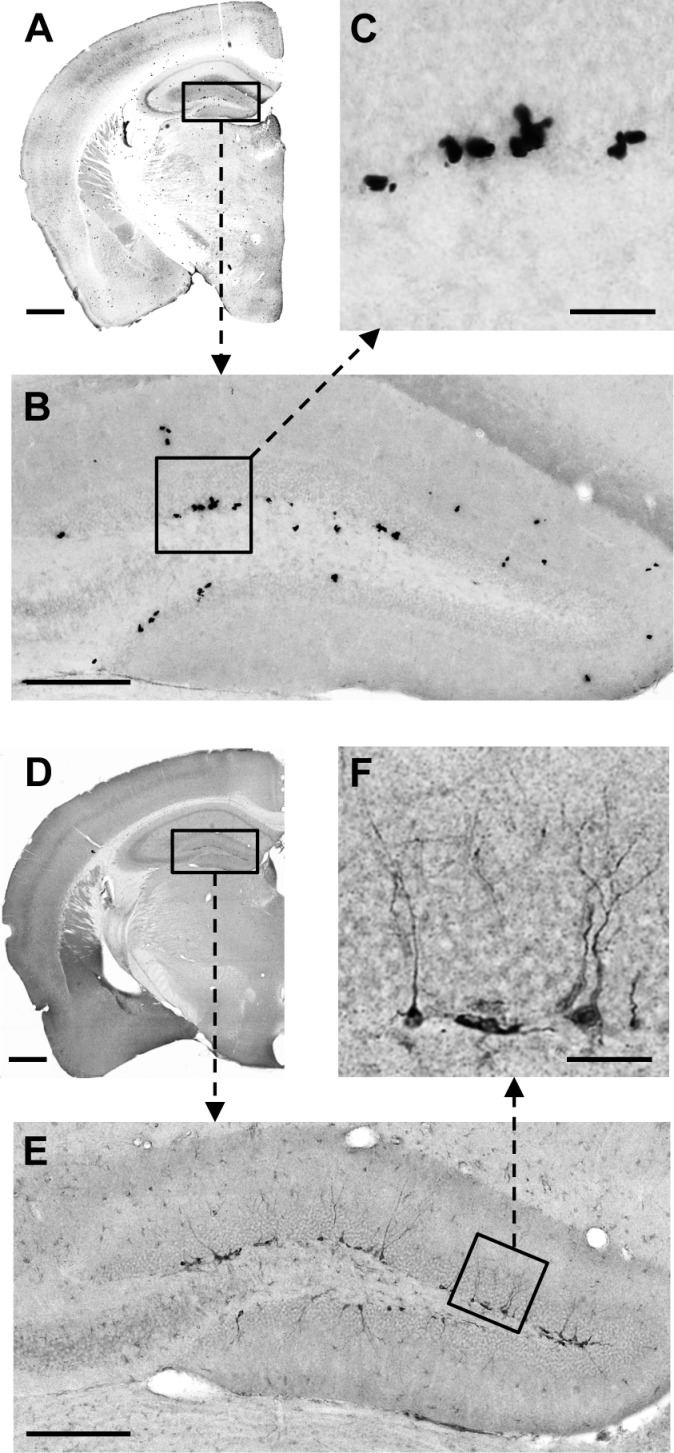
Representative coronal sections immunostained for BrdU and DCX quantification in the DG Low-magnification images (selected from −1.8 to −2.2 mm posterior to Bregma from an 8-month-old male WT mouse) demonstrate the distribution of BrdU (**A**) and DCX (**D**) immunoreactivity in a representative coronal sections. As predicted, the majority of BrdU +ve and DCX +ve cells were located in the hippocampus. Panels “**B**” and “**E**” are enlarged photomicrographs of the boxed areas from panels “**A**” (BrdU) and “**D**” (DCX) respectively. Panels “**C**” and “**F**” are high-power images illustrating BrdU and DCX DG staining patterns in more detail. The broken-line arrows point to the corresponding zoomed pictures. Scale bar: for **A** and **D** is 500 μm; for **B** and **E** is 150 μm; for **C** and **F** is 25 μm).

**Figure 2:**
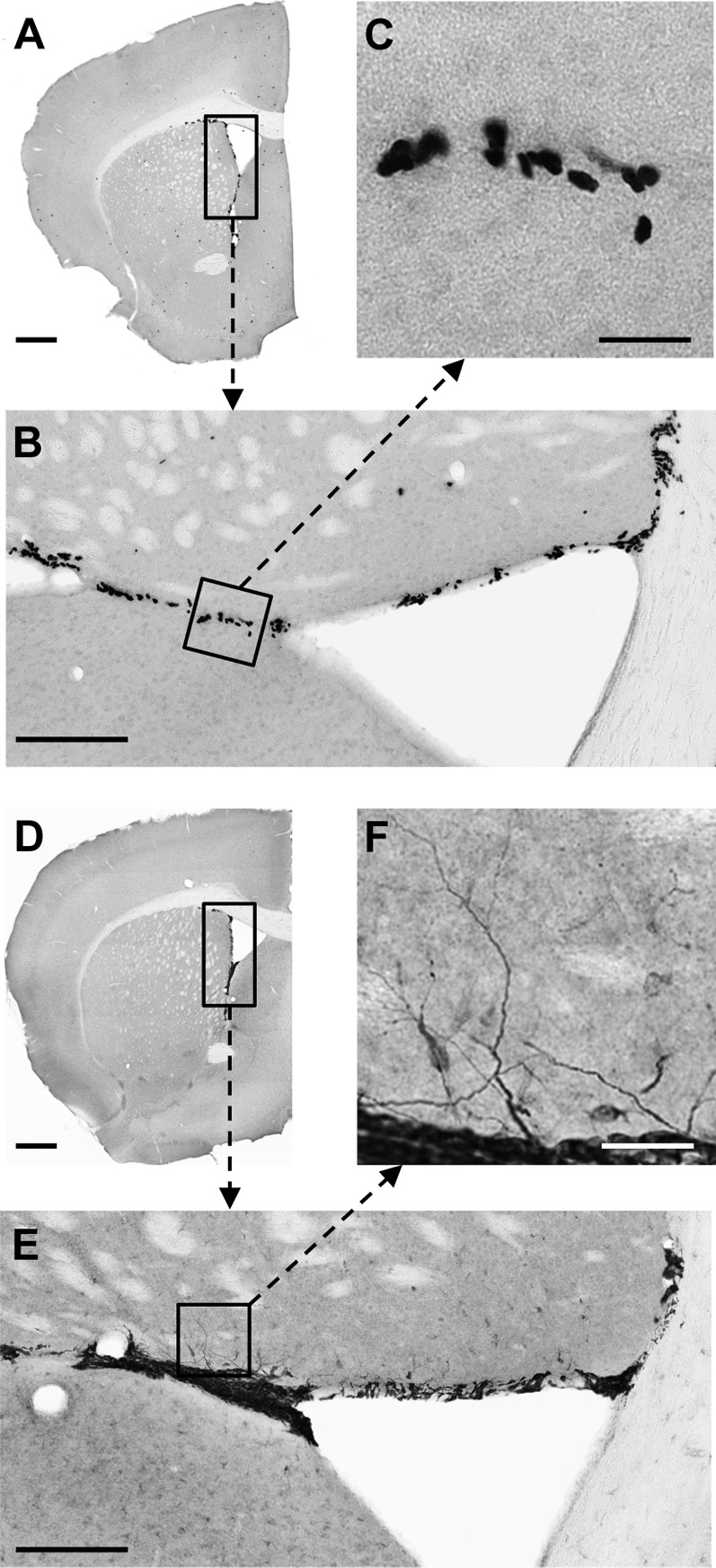
Representative coronal sections immunostained for BrdU and DCX quantification in the SVZ Low-magnification images [selected from +1.1 mm anterior to Bregma from an 8-month-old male *Abca7*^−/−^ mouse (**A**–**C**) and an 8-month-old male WT mouse (**D**–**F**)] demonstrate the distribution of BrdU (**A**) and DCX (**D**) immunoreactivity in representative coronal sections. Proliferating BrdU +ve cells were clearly detected within the wall of the lateral ventricle as illustrated in “**A**”, and viewed at higher magnifications (rotated 90° clockwise) in “**B**” and “**C**”. DCX staining of neuroblasts in a coronal section of the SVZ is presented in panels “**D**”–“**F**”. Fine processes with bifurcations were detected as DCX +ve immunostaining (in panel **F**) in the SVZ. Similar neuroblast morphology was seen in the DG (i.e. compare figure F with Figure 1F). The broken-line arrows point to the corresponding zoomed pictures. Scale bar: for **A** and **D** is 500 μm; for **B** and **E** is 150 μm; for **C** and **F** is 25 μm).

**Figure 3:**
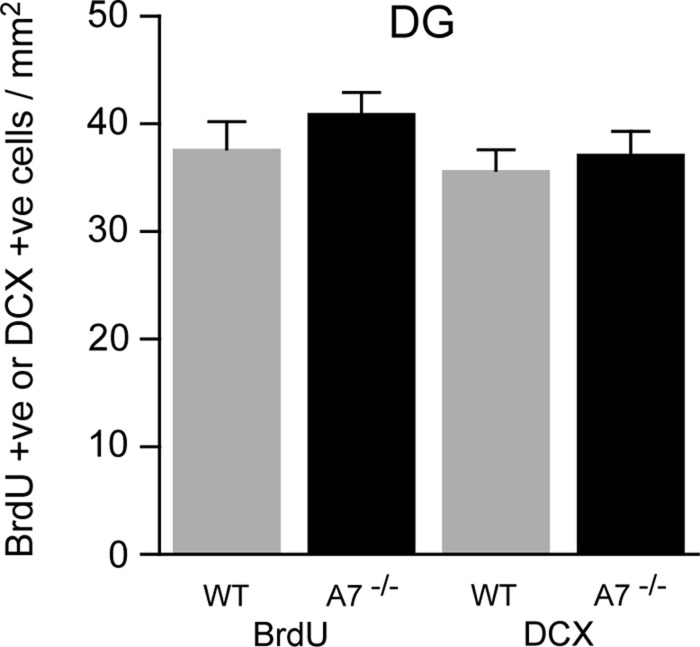
Cell counting in the DG confirms that deletion of *Abca7* does not have a significant impact on adult neurogenesis Histograms illustrate the quantitative evaluation of BrdU +ve cells and DCX +ve cells in the DG from WT and *Abca7*^−/−^ (A7^−/−^) mice. Measurement of the BrdU +ve and DCX +ve immunoreactive cell number was undertaken on five sections for in each mouse as described in the “Materials and Methods” section. The number of mice analysed was WT (*n*=7) and A7^−/−^ (*n*=7). There were no statistical differences in the number of either BrdU +ve or DCX +ve cells present in the DG when WT and *Abca7*^−/−^ animals were compared. Data (mean±S.E.M.) in histograms represent the number of cells detected/mm^2^. Note: due to the overlapping clusters of cells present in the SVZ (see Figure 2) and the difficulty in unambiguously identifying single cells, cell numbers were not quantified for this region.

**Figure 4:**
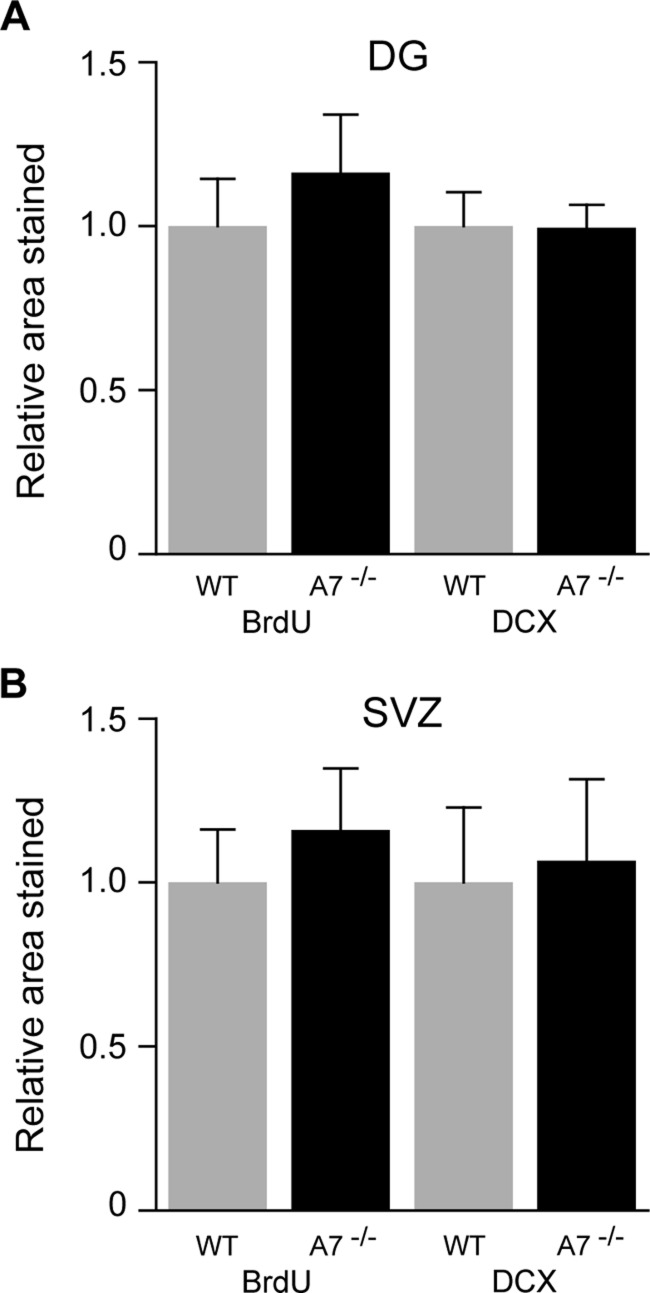
Deletion of *Abca7* does not have a significant impact on adult neurogenesis Histograms illustrate the quantitative evaluation of BrdU and DCX in the DG (**A**) and SVZ (**B**) from WT and *Abca7*^−/−^ (A7^−/−^) mice. Measurement of the positive BrdU and DCX immunoreactive area in specified regions of interest of both the DG and SVZ was undertaken on five sections for each region in each mouse as described in the “Materials and Methods” section. The staining analysis was quantified by ImageJ software. The number of mice analysed was WT (*n*=7) and A7^−/−^ (*n*=7). There were no statistical differences in the area (μm^2^/section) for BrdU or DCX immunoreactivity in either the DG or SVZ when WT and *Abca7*^−/−^ animals were compared. Data (mean±S.E.M.) in histograms represent the relative value as normalized with to WT (arbitrarily set at 1.0).

BrdU staining was used to assess cell proliferation in the DG of both WT and *Abca7*^−/−^ mice, whereas DCX staining was used as a marker of neurogenesis. Consistent with previous studies, we detected strong BrdU and DCX staining in the hippocampus (Figure 1). The immunohistochemical labelling was detected mainly in the subgranular zone of the DG. Evidence for neurogenesis was also clearly detected in the SVZ (Figure 2). Active cellular proliferation in the SVZ was detected by BrdU cluster-like staining within the wall of lateral ventricle (Figure 2E). Fine neuronal processes with bifurcations were evident in the SVZ when DCX immunostaining was applied (Figure 2F). We did not observe any gross (qualitative) differences to overall structure of proliferating cells when WT and *Abca7*^−/−^ mice were compared. Direct counting of BrdU +ve cells and DCX +ve cells in the DG indicated that *Abca7*^−/−^ loss did not significantly affect neurogenesis is this location (Figure 3). Furthermore, a quantitative evaluation of BrdU and DCX staining area in the DG and SVZ indicated there were no significant differences in neurogenesis comparing WT and *Abca7*^−/−^ mice (Figure 4).

Although we cannot rule out the possibility that glial progenitors are affected by *Abca7* loss, this seems unlikely given the lack of significant change in BrdU incorporation in both the DG and SVZ. Also, our findings do not preclude the possibility that ABCA7 contributes to embryonic neurogenesis as this has not been assessed in the present study.

There is mounting evidence that ABCA7 plays a role in regulating the pathways leading to AD [29]. Studies in cell lines and mouse models suggest that a loss of ABCA7 function may have deleterious consequences for Aβ homoeostasis in the brain [12–14]. This is consistent with the previously reported significant association of *ABCA7* SNPs with amyloid plaque load in human subjects [6]. Indeed, recent genetics studies have shown that loss-of-function *ABCA7* variants confer increased AD risk [10]. Even though the available evidence points towards a role for ABCA7 regulating Aβ homoeostasis, the true function of ABCA7 in the brain is still an open question. It is therefore important to investigate additional pathways that may be influenced by ABCA7 in order to understand its true function(s) in the AD context. An additional rationale for the present study is the literature suggesting certain ABC transporters play a role in the regulation of neurogenesis. In this context, ABCA2, ABCA3, ABCB1 and ABCG2 have been suggested to play a role in neurogenesis [15]. Furthermore, ABCA2 is a marker of neural progenitors in the adult mouse brain [16]. Despite the structural similarities of ABCA7 with these proteins, and the fact that ABCA7 is expressed at a relatively high level in the brain [2], the results from our current study suggest that ABCA7 does not play a major role in the regulation of cell proliferation or neurogenesis in the DG or SVZ of adult mice.

It is also worth considering how loss of ABCA7 function may impact adult neurogenesis in the AD setting. Although the roles that APP proteolytic fragments may play in regulating neurogenesis are far from clear [17–20,30,31], there is evidence that Aβ42 may inhibit adult neurogenesis [32–34]. Indeed, as previously suggested [17], the majority of AD mouse models that are based on APP mutations exhibit reduced neurogenesis. It might therefore be predicted that in an environment where ABCA7 is required to remove a toxic insult (e.g. phagocytic clearance of Aβ peptides that would otherwise inhibit neurogenesis), an indirect effect due to the loss of ABCA7 function may become apparent. These types of hypotheses are difficult to test experimentally as it would be hard to separate the effects of the toxic insult from ABCA7 function *per se*. Nonetheless, it remains possible that ABCA7 may have an impact on neurogenesis under certain conditions that were not modelled in our current assessment. Based on our current data, however, it is reasonable to assume that ABCA7 is unlikely to play a major direct role in adult neurogenesis in humans. In conclusion, our current BrdU incorporation and DCX labelling experiments indicate that deletion of *Abca7* does not have a significant impact on adult neurogenesis in mice.

## Abbreviations

Aβ: amyloid-β peptide
*Abca7^−/−^*: *Abca7* gene knockout mouse
ABCA7: ATP-binding cassette transporter, subfamily A, member 7
AD: Alzheimer's disease
APP: amyloid-β precursor protein
BrdU: bromodeoxyuridine
DCX: doublecortin
DG: dentate gyrus
PFA: paraformaldehyde
SNP: single nucleotide polymorphism
SVZ: subventricular zone
WT: wild-type-like
